# Co-Bonded Hybrid Thermoplastic-Thermoset Composite Interphase: Process-Microstructure-Property Correlation

**DOI:** 10.3390/ma14020291

**Published:** 2021-01-08

**Authors:** Jamal Seyyed Monfared Zanjani, Ismet Baran

**Affiliations:** Faculty of Engineering Technology, University of Twente, 7500AE Enschede, The Netherlands

**Keywords:** polymer interdiffusion, co-bonding, interphase, thermoplastics, thermosets curing, microhardness, hybrid composites

## Abstract

Co-bonding is an effective joining method for fiber-reinforced composites in which a prefabricated part bonds with a thermoset resin during the curing process. Manufacturing of co-bonded thermoset-thermoplastic hybrid composites is a challenging task due to the complexities of the interdiffusion of reactive thermoset resin and thermoplastic polymer at the interface between two plies. Herein, the interphase properties of co-bonded acrylonitrile butadiene styrene thermoplastic to unsaturated polyester thermoset are investigated for different processing conditions. The effect of processing temperature on the cure kinetics and interdiffusion kinetics are studied experimentally. The interphase thickness and microstructure are linked to the chemo-rheological properties of the materials. The interdiffusion mechanisms are explored and models are developed to predict the interphase thickness and microstructure for various process conditions. The temperature-dependent diffusivities were estimated by incorporating an inverse diffusion model. The mechanical response of interphases was analyzed by the Vickers microhardness test and was correlated to the processing condition and microstructure. It was observed that processing temperature has significant effect on the interdiffusion process and, consequently, on the interphase thickness, its microstructure and mechanical performance.

## 1. Introduction

Advanced fiber-reinforced polymeric composites have emerged as structural materials with a very wide range of applications due to their significant weight reduction, extended durability, and higher design flexibility compared to other traditional material alternatives. However, there are ever-pressing industrial needs for further improving the performance of polymeric composites and to meet the requirements of demanding and cutting edge applications. Combining various advanced materials and creating hybrid or multi-material composites (MMCs) have the potential to push forward the mechanical performance of the manufactured part. The laminated MMCs consist of two or more different combinations of layers, which offer a design versatility to place each specific material, where they can exploit their best characteristics and properties to the benefit of the overall integrity of the structure. Hence, it is considered that the laminated MMCs have the potential to develop future structural materials with high performance [1,2]. Thermoplastic-thermoset hybrid polymer-based composites are one of the possibilities of MMCs with significant potential to address challenges for many state of the art applications [3]. Thermoplastic-thermoset MMCs combine the damage tolerance characteristics, and welding capability of thermoplastics with the strength and stiffness of thermoset composites resulting in more reliable structures with an extended lifetime [4,5,6]. However, joining thermoplastics to thermosets remains a challenging step despite the development of various joining technologies for composite structures including (i) mechanical fastening [7,8,9], (ii) welding [10,11,12,13], (iii) adhesive bonding [14,15,16], (iv) co-curing (parts are cured at the same time) [17,18], and (v) hybrid joints (combination of two or more of previous methods) [19,20,21]. Nevertheless, all these joining methods have disadvantages in terms of being unreliable, labour-intensive, time-consuming and expensive [22].

An alternative and effective joining method for composites is co-bonding (an uncured part is joined with one or more cured parts) [23,24]. The co-bonding method can be applied for jointing both similar or dissimilar materials including thermoplastics to thermosets. Co-bonding eliminates the needs for mechanical fasteners, and adhesives resulting in a more effective and applicable joining method for many applications. The interface between two parts in the co-bonding process is controlled by thermodynamic affinity and physical interactions in between and also the curing process of the reactive resin [10]. Several studies have been conducted on co-bonding process of thermoplastic-thermoset for epoxy thermoset resins and various thermoplastics. In [25], the difference in diffusion of a bisphenol A type epoxy resin and the corresponding diamine curing agent into polysulfone (PSU) was studied. The diffusivity of amine was found to be an order of magnitude larger than that for epoxy at different temperatures. As an extension to the aforementioned study, Rajagopalan et al. [26] showed that the amine enhanced the epoxy diffusivity into PSU by a factor of three due to amine swelling effect. In addition, they identified three chronological processes of diffusion, reaction, and phase separation which derive the interphase formation. In another study, Sonnenfeld et al. [27] improved the damage tolerance of epoxy matrix composites through manufacturing of thermoplastic/thermoset multilayer composites using semi-crystalline poly-ether-ether-ketone (PEEK) and polyphenylene sulfide (PPS). They demonstrated that the introduction of a layer which was compatible with both thermoplastic and thermoset sections such as poly-ether-imide (PEI) increased the adhesion energy by a factor of 15. Velthem et al. [28] studied the influence of the interdiffusion of two thermoplastics of poly(ether sulfone) (PES) and phenoxy in the epoxy system and they correlated the resulting interphase morphologies to the interlaminar fracture toughness. Nevertheless, all these studies were limited to epoxy as the thermoset resin and focused on the interdiffusion process and morphology of interphase.

Processing condition plays an essential role in interphase formation between thermosets and thermoplastics during co-bonding by altering the cure reaction kinetics, and interdiffusion process. The curing reaction of a thermosetting resin is a multi-stage complex and highly temperature-dependent process. In the curing of a thermoset resin, many reactions take place simultaneously such as the decomposition of the initiator, the polymerization of different monomers and oligomers, and termination reactions with changes in the physical behaviour of the resin. All these reactions influence the interdiffusion process and interphase formation since the degree of cure or degree of cross linking of the molecules increases as a function of process time and temperature as shown in [29,30]. Therefore, the interdiffusion kinetics and, consequently, the interdiffusion termination time as determining parameters on the interphase formation are closely linked to curing kinetics of the thermoset resin [31]. More specifically, as the cure reaction progresses the molecular weight and crosslinking density of the resin increase after which no polymer interdiffusion takes place. Regarding the cessation of the polymer interdiffusion at the thermoset-thermoplastic interface, the most accepted assumption is that diffusivity of the reacting resins terminates at the gel point defined as a time in which the resin switches its state from a viscous liquid to a semi-solid rubbery gel and loses its fluidity [12,32,33,34]. Accordingly, the kinetics of curing and interdiffusion determines the interphase thickness, its quality, and the microstructure. It is known that the mechanical performance and strength of interphase in hybrid composites are primarily determined by the interphase thickness [35,36]. Therefore, a physical and mechanical understanding of the interphase is critical to determine the load transfer efficiency across dissimilar materials and to oversee the overall integrity of the structure. To the best of the authors’ knowledge, there is no comprehensive study elaborating on the effect of processing conditions and cure kinetics on the micro-structure and mechanical response of the interphases in co-bonded thermoplastic-thermoset parts in the literature.

In this study, unsaturated polyester resin (UPR) for the first time is used to be co-bonded to a thermoplastic part. UPR is one of the widespread used thermoset resins in various industry due to its low cost and excellent processability in many applications such as wind energy and ship manufacturing industries [37,38]. On the other hand, a terpolymer of acrylonitrile, butadiene, and styrene with commercial name of acrylonitrile butadiene styrene (ABS) known to have excellent mechanical properties including high toughness, ductility, and very high impact strength is selected as the thermoplastic material [39]. Herein, the effect of processing temperature on the co-bonding of ABS and UPR and resultant interphase morphology and mechanical response are investigated. The interdiffusion kinetics and mechanisms during co-bonding are explained and linked to the chemo-rheological properties of UPR. Models are developed and utilized to predict the interphase thickness at various processing conditions and to provide information on interphase microstructure. The experimentally characterized gel time and interdiffusion thickness are used in a one-dimensional (1D) inverse diffusion model to estimate the diffusivity at different temperatures. Subsequently, the diffusivities are fit to an Arrhenius type of model. Vickers’ microhardness test was used to study the mechanical behaviour at the interphase of the specimens at various temperatures. Finally, a methodology is provided to assemble process-microstructure-property correlations at interphases which are essential to the design and manufacturing of reliable thermoplastic -thermoset MMCs. 

## 2. Materials and Methods

### 2.1. Materials

An industrial medium reactive orthophthalic unsaturated polyester resin (UPR) designed for resin transfer moulding and vacuum injection moulding processes as described in [40] was used as thermoset resin. This resin contained 45% styrene with an acid number of 25 mgKOH/g. A liquid peroxide system specialized for curing of the unsaturated polyester and vinyl ester resins at room temperature with low peak exotherm, and long working time (gel time) was used as an initiator. ABS in the sheet form with grade name of VIKUREEN ABS PLAAT GLANS WIT 0291 (Epsotech, Jülich Kirchberg, Germany), with strong resistance to corrosive chemicals and/or physical impacts, was used as thermoplastic material in this study.

### 2.2. Interphase between Thermoset and Thermoplastic

**Figure 1:**
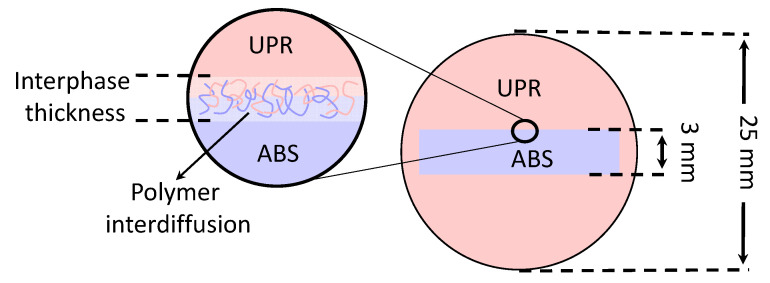
Schematic view of the interphase region in the acrylonitrile butadiene styrene-unsaturated polyester resin (ABS-UPR) co-bonded specimen [31].

To study the interdiffusion of thermoset resin into thermoplastic, ABS specimens were prepared by cutting them using a wet saw into parts with estimated dimensions of 15 mm × 15 mm × 3 mm followed by edge sanding with #500 SiC foil to remove any debris. Next, an isopropyl alcohol and a mixture of water were utilized to wash ABS specimens to remove any remaining particles and contamination. Specimens were dried in the oven at a temperature of 60 °C for 24 h and then left in a desiccator to reach room temperature. Afterward, ABS specimens were placed vertically in the middle of a cylindrical embedding mould with a diameter of 25 mm by using a couple of metallic clamping rings. The degassed UPR resin mixture was poured onto the ABS specimens until the resin completely covered them. Effect of temperature on the interphase was studied by preparing the samples and placing of the moulds in the preheated oven fixed at the set temperatures of 25 °C, 35 °C, 40 °C, 50 °C, and 60 °C and curing for 24 h followed by a post-curing process at 60 °C for 24 h. After the de-moulding of samples, Struers polishing system (Teramin-30, Struers, Cleveland, OH, USA) was employed to polish the cross-sections of specimens with SiC foil of #500, #1000, #2000 and #4000, in the given order. The final polishing of the embedded specimens were done by using OP-S NonDry. A schematic view of the cross-section for the co-bonded ABS-UPR sample and the resulting interphase due to polymer interdiffusion are shown in Figure 1.

### 2.3. Characterizations

#### 2.3.1. Microscopic Analysis of Unsaturated Polyester Resin-Acrylonitrile Butadiene Styrene (UPR-ABS) Interphase+

The polished cross-section of interphases formed between the co-bonded ABS and UPR systems at various processing conditions were analysed by using a Keyence VHX-5000 digital microscope (Keyence, Osaka, Japan) equipped with a VH-100UR lens. 2D stitching function of the microscope software was used to joint several captured images to obtain a sufficiently large cross-section of the interface. The interphase thickness values were reported for each temperature as the average measurements at mid-section of the interface far from the clamps and obtained from at least three specimens for each temperature.

#### 2.3.2. Surface Swelling Measurements

**Figure 2:**
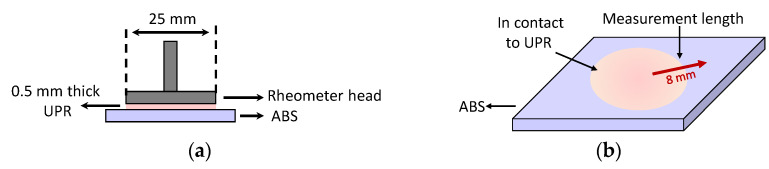
(**a**) Schematic view of the rheometer setup for the swelling experiments by placing UPR on ABS surface, and (**b**) measurement direction in confocal microscopy.

Furthermore, the surface swelling of ABS by UPR resin were examined at room temperature. For this, a parallel plate apparatus of the rheometer (in stationary mode) (Anton Paar Germany GmbH, Ostfildern, Germany) was employed to confine the resin between the head plate and ABS sheet (as the bottom plate) as shown in Figure 2a. The nominal normal force was set to 0.1 N in the rheometer. The normal force was kept constant to ensure the contact of resin to the rheometer head and surface of the specimen and to prevent resin starvation due to diffusion at the study surface. The capillary forces between UPR and the plates which might cause a possible resin flow out was prevented by the plate-plate configuration. Herein, 50 mm × 50 mm ABS plates were cut, edge sanded as mentioned in detail in Section 2.2, and then were glued on the surface of the bottom plate of the rheometer. The plate-plate distance was set to 0.5 mm and was filled with UPR, i.e., without mixing it with the liquid peroxide system, and kept for 60 min. Next, Keyence VK-9700 confocal microscope was used to capture the changes in the surface topology of ABS due to swelling by UPR. The height and spatial resolutions were 1 and 120 nm in the confocal microscope which had a laser with 408 nm wavelength. As seen from Figure 2b, the confocal microscopy measurements were performed on the path which covered the region with and without UPR to define the surface swelling of APB. The length of the scanned length by the confocal microscopy was approximately 8 mm.

#### 2.3.3. Chemo-Rheology of UPR

The chemo-rheological properties of the UPR were characterized by using the Anton Paar-Physica MCR 501 rheometer (Anton Paar Germany GmbH, Ostfildern, Germany) in “plate–plate” mode. The effect of processing temperature on the viscosity, storage modulus (G′) and loss modulus (G″) were investigated. All the measurements were performed by using circular aluminium plates of 25 mm diameter in oscillatory mode at a 0.5% strain and a 1 Hz with a plate-plate spacing of 0.3 mm. The temperature-dependent gel time of the resin, as an indication of the cure kinetics of UPR resin, was estimated from the crossing point of storage modulus (G′) and loss modulus (G″) of UPR mixture including the liquid peroxide [41]. The rheometer measurements were performed at seven different isothermal temperatures of 25 °C, 35 °C, 40 °C, 50 °C, 60 °C, 80 °C and 100 °C and final gel time at each temperature was reported as the average of at least three repetitions. In the gel-time measurements, the rheometer was maintained at the set temperature for 30 min before placing the resin to achieve a uniform and stabilised temperature in the measurement chamber. The relation between temperature-dependent gel time values and temperature was describe by employing an Arrhenius relationship as [42]:

(1) $$\left(\frac{1}{t_{G}}\right)=\left(\frac{1}{t_{0}}\right)exp\left(\frac{-\Delta E}{RT}\right)$$

where *t_G_* was gel time, *t*_0_ was the pre-exponential time constant, *R* was the universal gas constant, and Δ*E* is the activation energy of the gelation. Herein, gel time as an index of cure kinetics was employed to monitor the changes during the curing reaction and to determine the cure kinetics effect on interphase formation. The viscosity measurement was performed on the UPR (without initiator) with a ramp of 1.5 °C/min between room temperature to 85 °C where measurements were taken every 10 s and resulted in 240 measurement points.

#### 2.3.4. Diffusivity Coefficient of UPR in ABS

##### 1D Inverse Diffusion Model

**Figure 3:**
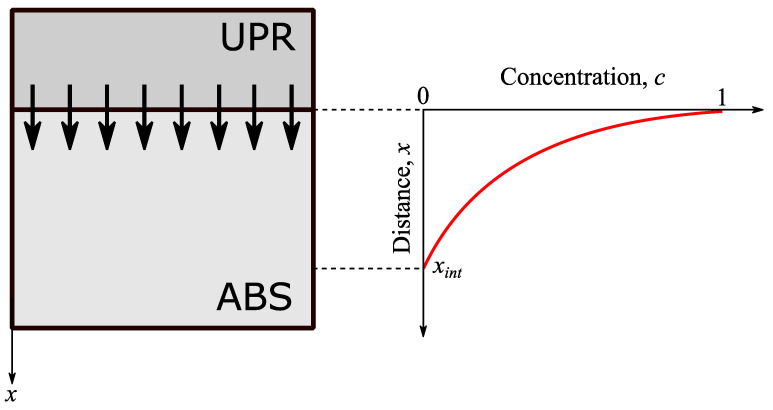
Schematic view of the 1D diffusion of UPR into ABS (**left**). The resulting concentration distribution (**right**).

The 1D interdiffusion of the UPR into ABS as schematically shown in Figure 3 was modelled using the Fick’s second law [43] as:

(2) $$\frac{\partial c\left(x,t\right)}{\partial t}=D\frac{\partial^{2}c\left(x,t\right)}{\partial x^{2}}$$

where c(x,t) was the UPR concentration in ABS, *D* was the diffusivity or coefficient of diffusion. By assuming *D* is constant at a certain temperature and the volume of ABS does not change with the diffusion of UPR into ABS, Equation (2) can be solved analytically as [44]:

(3) $$\frac{c\left(x,t\right)-c\left(0,t\right)}{c\left(x,0\right)-c\left(0,t\right)}=\mathrm{erf}\left(\frac{x}{2\sqrt{Dt}}\right)$$

where erf was the error function, c(0,t) was the concentration at x=0 and c(x,0) was the initial concentration. Since the interdiffusion distance ($x_{int}$) was experimentally determined, the corresponding D can be obtained from Equation (3) by considering the conditions in the following:

- c(0,t) = 1 for $\;t\leq t_{gel}$ and c(0,t) = 0 for $t>t_{gel}$ where $t_{gel}$ was the gelation time of the polyester resin which was determined experimentally in Section 2.3.3.
- The concentration was assumed to be relatively small by using a value of 10^−5^ at $t=t_{gel}$ and at $x=x_{int}$, i.e., $c\left(x_{int},t_{gel}\right)=10^{-5}$ (see Figure 3).

Accordingly, D was obtained using Equation (4). Note that D was estimated for different $t_{gel}$ and $x_{int}$ which were obtained at different process temperatures.

(4) $$D=\frac{1}{t_{gel}}{\left(\frac{x_{int}}{2{\mathrm{erf}}^{-1}\left(1-c\left(x_{int},t_{gel}\right)\right)}\right)}^{2}$$

##### Temperature Dependent Diffusivity Model

The obtained D values from the 1D inverse diffusion model for different temperatures were fit with an Arrhenius type of equation as follows [44]:

(5) $$D=D_{0}\exp\left(\frac{-E_{1}}{RT}\right)$$

which was the diffusivity model also used in [25,26,44]. In Equation (4), *D*_0_ was the pre-exponential constant, *E*_1_ was the activation energies, *R* was the universal gas constant and *T* was the temperature in K. A linear regression was employed for the fitting procedure by transforming the non-linear relation in Equation (5) into a linear one as:

(6) $$\ln\left(D\right)=\ln\left(D_{0}\right)-\frac{E_{1}}{RT}$$

#### 2.3.5. Resin Uptake

The kinetics of the interdiffusion was investigated by resin uptake experiments where ABS specimens were immersed into a UPR bath (without initiator) and the weight of the absorbed resin by specimens were measured at different times of 60, 300, 600, 1200, 1800, 3600 and 7200 s after immersion and temperatures of 25 °C, 35 °C, 40 °C, 50 °C, and 60 °C. The nominal dimensions of the ABS specimens were 30 mm × 30 mm which were measured by using a micrometre with a precision of ±0.01 mm. An analytical balance was used to measure the dry weight of specimens. The immersion of the specimens in to the resin was carried out by using the metallic clamps. The excess resin on the specimen surface after the immersion step was washed by using an ethanol. In addition, non-sticking and absorbent fabrics were used to clean the specimen surfaces. After drying, resin uptake weight were measured by the analytical balance and the uptake values per contact area (M*) were calculated using the below formula:

(7) $$M^{*}=\frac{W_{i}-W_{d}}{A}$$

where *W_i_* and *W_d_* were the resin uptake weight and dry ABS specimen weight, respectively, M* was the mass uptake per unit area and A was the surface area. As the length and width of specimens were considerably (over 10 times) larger than the thickness, the resin uptake from the edges of ABS samples was neglected. The measured data were fitted to Equation (8) in order to evaluate the transport type and associated diffusion kinetics (resin uptake) [45].

(8) $$M^{*}=kt^{n}$$

where *k* was an empirical rate constant, *t* was the time and *n* was the transport exponent of diffusion. The type of diffusion is indicated by n in Equation (8), i.e., *n* = 0.5 for Fickian diffusion, 0.5 < *n*<1 for an anomalous diffusion, and *n* = 1 for Case II diffusion [45].

Nevertheless, to obtain a kinetics model for a continuous range of temperatures based on Equation (8), it was needed to have a model for *n* and *k* over experimental temperatures. Herein, *n* and *k* are approximated by Equations (9) and (10) as:

(9) $$n=A_{n}exp\left(\frac{N_{0}}{T}\right)$$

(10) $$k=k_{1}+k_{2}T$$

where *A_n_*, *N*_0_, *k*_1_, *k*_2_ were defined as constants to be determined through fitting of experimental results.

#### 2.3.6. Resin Volume Fraction at Interphase

To correlate the diffusion kinetics to resin curing kinetics and consequently interphase formation, Equations (1) and (8)–(10) are combined into Equation (11). This eliminated the time term from resin uptake equation (Equation (8)) for the reactive resin and yield to a model for amount of diffused reactive resin per unit area (M*) into ABS at given temperature.

(11) $$M^{*}=\left(k_{1}+k_{2}T\right){\left(\frac{t_{0}}{exp\left(\frac{-\Delta E}{RT}\right)}\right)}^{\left(A_{n}\exp\left(\frac{N_{0}}{T}\right)\right)}$$

Moreover, the relation between M* and *V_r_* (the volume of the diffused resin) was obtained by using the following relation:

(12) $$M^{*}=\frac{M}{A}=\frac{\rho V_{r}}{A}=\rho V_{r}^{*}$$

where *ρ* and *A* were the density of uncured resin (1.05 gr/cm^3^) and contact surface area, respectively. Herein, V_r_* was defined as Vr*=VrA and so-called reduced resin volume (diffused volume per unit of contact area). V_r_* values at any given temperature were obtained from combining Equations (11) and (12) as:

(13) $$V_{r}^{*}=\left(\frac{1}{\rho }\right)\left(k_{1}+k_{2}T\right){\left(\frac{t_{0}}{exp\left(\frac{-\Delta E}{RT}\right)}\right)}^{\left(A_{n}\exp\left(\frac{N_{0}}{T}\right)\right)}$$

Accordingly, the ratio of V_r_* to reduced volume of interface V_int_* which corresponds to interphase thickness provided the volume fraction of resin at the interphase (*φ*) as an essential microstructure indicator in binary interphases defined as:

(14) $$\varphi =\frac{V_{r}^{*}}{V_{int}^{*}}$$

#### 2.3.7. Hardness Measurement at Interphase

**Figure 4:**
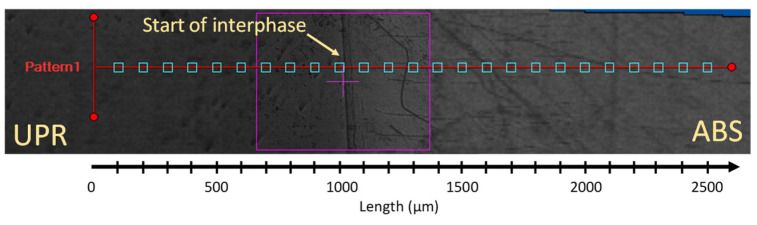
Vickers microhardness measurement pattern and sample configuration.

The Vickers microhardness test was employed to analyse the changes in the mechanical response at the UPR-ABS interphase. The hardness obtained by this method was described as the resistance of the surface to indentation [46]. Vickers microhardness mapping was conducted using the LECO LM100AT micro-hardness tester by applying a load of 10 gr and an indent spacing of 100 µm. The Vickers diamond pyramid hardness number, *H_V_*, was defined as the ratio of the applied load, *P*, to the pyramidal contact area of the indentation:

(15) $$H_{v}=\alpha \frac{P}{d^{2}}$$

where *d* was the diagonal length of the resultant impression, and *α* was defined as 1.8544 for the Vickers indenter [47]. Figure 4 shows an example pattern and sample configuration used in the microhardness measurements in the vicinity of interphase for ABS and UPR. A total of 25 indentations for each line were conducted with 100 µm intervals in the width of interphase as shown in Figure 1 in which the initial 1000 µm was for the UPR and the rest covered the interphase and ABS regions. The hardness profiles were reported for each temperature determined from the average measurements for at least three specimens.

## 3. Results and Discussion

### 3.1. Interphase Formation and Processing Temperature Effect

**Figure 5:**
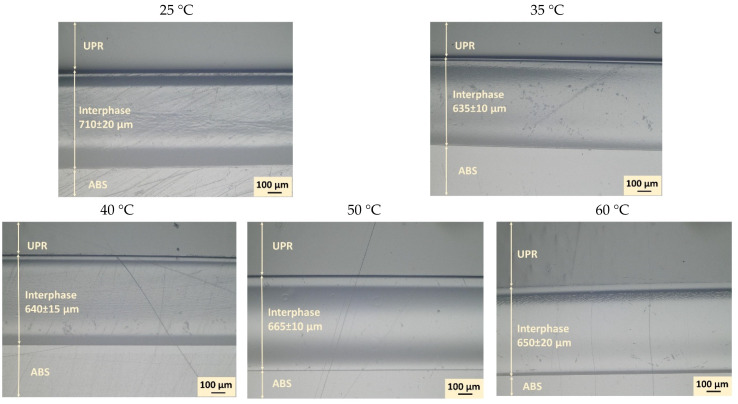
Micrographs showing the interphase formation between ABS and UPR at different temperatures.

**Figure 6:**
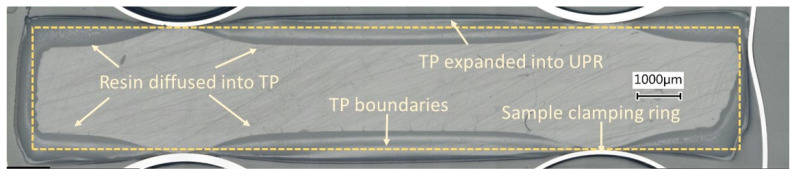
Overall view of the interphase formed between ABS and UPR at 25 °C.

**Figure 7:**
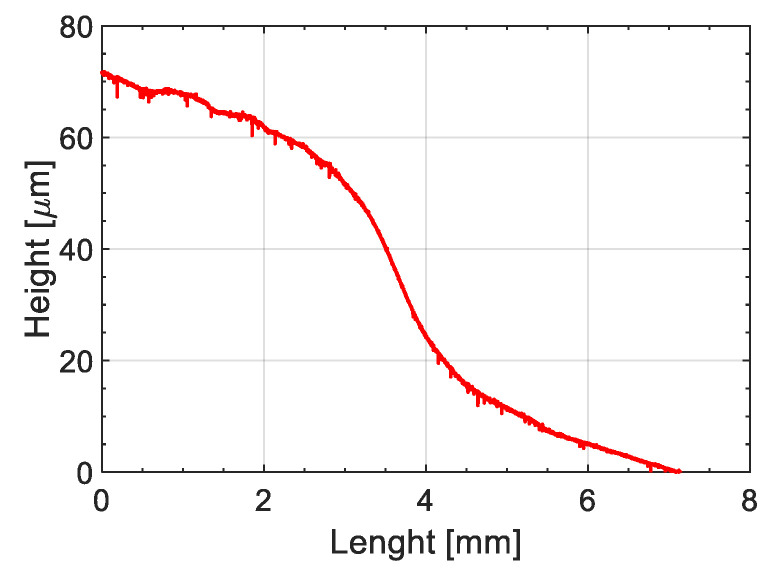
Confocal microscopy measurement of surface swelling of ABS in contact with UPR.

Figure 5 reveals the optical microscopic observations of ABS-UPR interphases indicating the average interphase thickness formed at different temperatures ranging from 25 °C to 60 °C. It was observed that an interphase was formed between ABS and UPR at all temperature ranges with no visible gradient of diffusion. Nonetheless, the interdiffusion fronts in both sides of formed interphases were slightly different from inner sections which implies the contribution of various phenomena in interphase formation. Therefore, an overview image of interphase formed around the ABS specimen at 25 °C was constructed by stitching several overlapping microscopic images as shown in Figure 6 to illustrate the effect of boundary conditions and constraints such as metallic clamps on the interphase formation. In Figure 6, a dash-lined frame is inserted in the image to estimate the boundaries of the original ABS specimen before interactions with UPR. The frame size and location were approximated, based on the location of metallic clamping rings and nominal thickness of the ABS sample before embedding. Figure 6 reveals that the interphase formed around the ABS noticeably exceeded the boundaries of the original ABS sample (dashed frame). To elaborate, it was expected to have the diffusion in both directions of UPR into ABS and ABS into UPR. Nevertheless, diffusion of ABS into UPR was at a much lower level compared to UPR into ABS due to the higher molecule size and lower molecular mobility of ABS as compared with liquid UPR [48,49]. However, the difference in the interphase thicknesses inside and outside of the frame was not as significant as it was anticipated. Therefore, swelling of ABS with UPR was a contributing parameter in the interphase thickness. In this regard, the swelling behaviour of ABS in contact with UPR was studied by placing the UPR on the surface of the ABS sample in the rheometer as mentioned. Figure 7 depicts the confocal microscopy results of the surface profile of ABS after being in contact with UPR in the rheometer. It is clearly seen that the ABS surface was swelled and its height increased approximately 70 μm as compared with the rest of the specimen. The increase in the ABS sheet thickness confirmed the swelling behaviour of ABS in contact with UPR.

**Figure 8:**
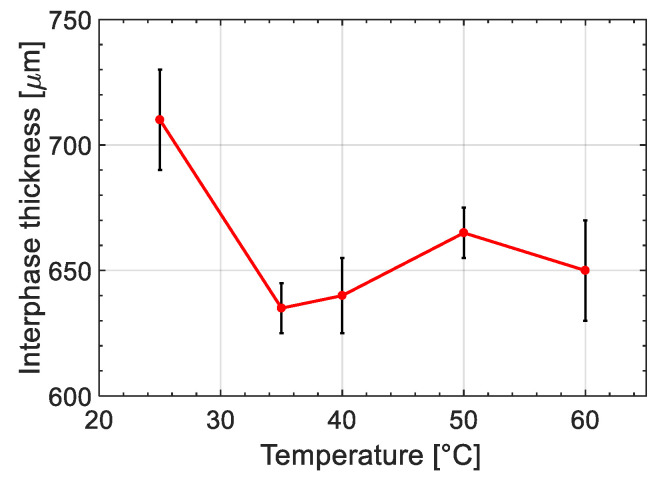
Interphase thickness formation between ABS and UPR for different processing temperatures.

Moreover, it can be seen in Figure 5 that the process temperature influenced the interphase thickness considerably. Figure 8 displays the changes in interphase thickness with respect to the process temperature. It is seen that the thickest interphase was achieved for specimen prepared at 25 °C with an interphase thickness of 710 ± 20 μm while the thinnest interphase was observed at 35 °C as 635 ± 10 μm. It can be seen in Figure 8 that the interphase thickness sharply decreased by increasing the temperature from 25 °C to 35 °C followed by a gradual increase after 35 °C up to 50 °C and slightly drops from 50 °C to 60 °C. The controversy effect of temperature on interphase thickness under and above 35 °C can be described by the effect of temperature on the cure kinetics and diffusion kinetics of the resin. An increase in temperature was expected to accelerate the curing kinetics leading to a decrease in the time available for the interdiffusion process (decreasing factor for interphase thickness). However, the rate of change in the cure kinetic is different at low and high temperatures. At higher temperature, the peroxide was already over its thermal activation temperature threshold (the employed initiator was designed for application in room temperature and above it). Therefore, the acceleration rate was lower while at the lower temperature an increase in temperature can provide the necessary activation energy for peroxides and boost the reaction kinetics significantly [50]. To obtain a comprehensive understanding of the effect of temperature on the interphase formation, cure kinetics and its mechanical properties, and interdiffusion process were investigated and further described in the next section.

### 3.2. Resin Curing and Diffusion Kinetics

**Figure 9:**
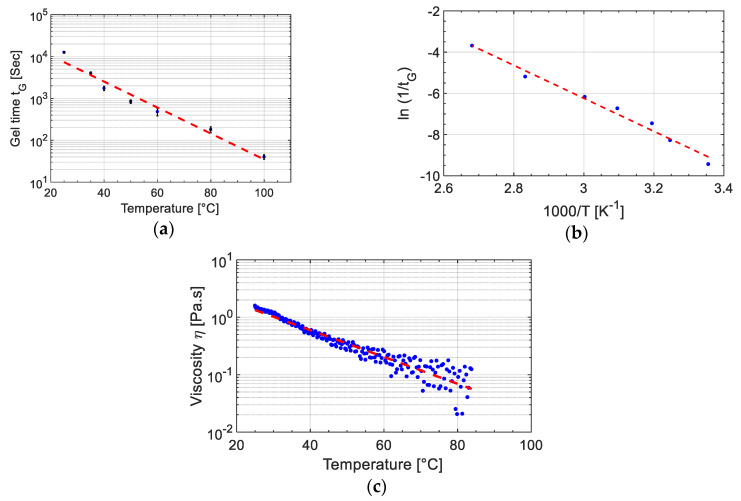
(**a**) change in gel time vs. temperature, (**b**) ln(1/*t_G_*) vs. 1000/*T* and fitted line based on Equation (16), and (**c**) viscosity change as a function of temperature for UPR.

Figure 9a shows the gel time obtained with respect to isothermal rheometer tests. It is seen that the gel time changed linearly in the logarithmic scale with temperature indicating that the gel time was considerably shortened by an increase in temperature. Herein, Equation (1) is reorganized as Equation (16) to determine the constants from the fit line in the graph ln(*t_G_*) vs. 1000/*T* graph as presented in Figure 9b.

(16) $$\ln\left(\frac{1}{t_{G}}\right)=\ln\left(\frac{1}{t_{0}}\right)-\Delta E/RT$$

The slope and intercept for Equation (16) of the fit line were calculated to be −8.005 K and 17.77, respectively, which correspond to the pre-exponential time constant of 2 × 10^−8^ s and activation energy of 66.5 kJ/mol.

Moreover, the changes in the temperature were associated with the alterations in interdiffusion kinetics by increasing the molecular mobilities indicated by a reduction in the viscosity of the resin as seen in Figure 9c [28,51]. In Figure 9c, it was found that the viscosity of UPR changed linearly with temperature in logarithmic scale and a small increase in temperature reduces the viscosity considerably which can promote the interdiffusion and result in thicker interphase. It is worth noting that to eliminate the effect of curing on the viscosity of the resin, initiator was not added to the resin in viscosity measurement tests [30].

These observations regarding the changes in cure kinetics via the gel time and resin viscosity by temperature clearly described the drop in the interphase thickness between 25 °C and 35 °C, i.e., the gel time decreased significantly in this region. On the other hand, after 35 °C the change in gel time took place at much lower levels. To elaborate, the gel time dropped approximately from 12500 s at 25 °C to 4000 s at 35 °C. This indicated that 8500 s less time was available for the polymer diffusion. The gel time difference between 40 °C and 50 °C was only about 900 s and it further declined from 50 °C to 60 °C as the gel time difference was obtained as approximately 360 s. On the other hand, at higher temperatures viscosity of the resin dropped to much lower values compared to the viscosity at lower temperatures. This low viscosity at high temperatures promoted the interdiffusion process with limited change in the gel time leading to a gradual increase in the interphase thickness between 35 °C to 50 °C. Furthermore, the significantly shorter gel time at 60 °C compared to 50 °C is the reason to its lower interphase thickness.

### 3.3. Diffusivity Coefficient and Model

**Figure 10:**
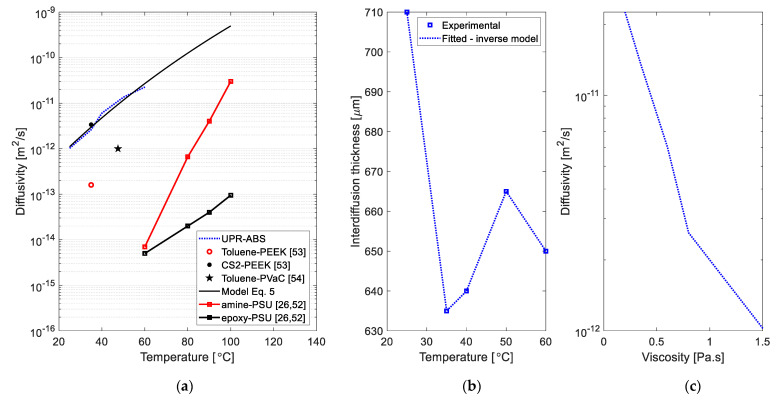
(**a**) Diffusivity vs temperature for UPR-ABS in comparison to material pairs from literature, (**b**) interphase thickness change by temperature through fitted inverse model, and (**c**) viscosity dependency of the diffusivity for UPR-ABS.

The diffusivity of UPR into ABS was estimated by using Equation (4) and the results obtained are shown in Figure 10. The inverse diffusion model resulted in an exact value for the interdiffusion thickness as seen in Figure 10b for the corresponding diffusivity values. It can be seen in Figure 10a that the fitted diffusivity was found to be in the range of 10^−12^–10^−10^ (m^2^/s) for the temperature range of 25–60 °C. The estimated magnitudes of the diffusivities matched with the reported diffusivities of other systems such as epoxy-PSU [26,52], amine-PSU [26,52], toluene-PEEK (for 0% initial crystallinity of PEEK) [53], CS2-PEEK (for 0% initial crystallinity of PEEK) [53] and toluene-PVAc (for mass fraction of toluene of 0.1) [54] as seen in Figure 10a. The trend of the temperature dependent diffusivity was captured well with the temperature dependent model given in Equation (5) with $D_{0}=1.69\times 10^{1}$ m^2^/s and $E_{1}=7.52\times 10^{4}$ cal/mol. The diffusivity coefficient magnitude obtained and its trend with temperature was in conformity with available literature. The relation between the experimentally obtained viscosity and the temperature dependent diffusivity obtained by the inverse diffusion model is shown in Figure 10c. It can be seen that the diffusivity was found to be inversely proportional which supported the aforementioned observations regarding the interdiffusion thickness and viscosity. In addition the trend obtained for the diffusivity vs viscosity also matches with the findings in [55] for the diffusion of organic molecules in sucrose–water solutions.

### 3.4. Kinetics of Interdiffusion and Its Correlation to Interphase Microstructure

**Figure 11:**
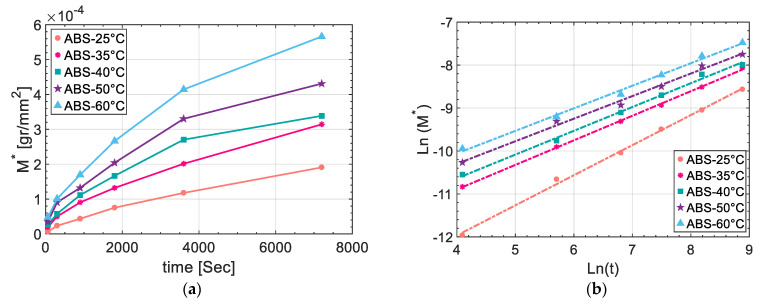
(**a**) Resin uptake vs. time for ABS, and (**b**) Ln(M*) vs. Ln(*t*) based on Equations (7) and (8).

**Figure 12:**
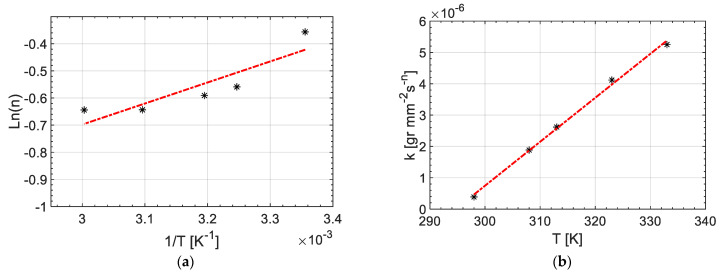
Line fitting on (**a**) n and (**b**) *k* changes by temperature to obtain predictive models based on Equations (9) and (10).

Figure 11a depicts the resin uptake per surface area (M*) vs. time at the different temperatures ranging from 25 °C to 60 °C. It can be seen that increasing the temperature enhanced the resin uptake value for all the measurement times. A line fitting was employed for the relation between Ln (M*) and Ln (*t*) seen in Figure 11b to determine *k* and *n* (see Equation (8)) and the results obtained are listed in Table 1. It was observed that n value changed with a change in temperature however it remained between 0.5 and 1 which indicated the anomalous nature of the diffusion process. More specifically, the value of n in Equation (8) sharply dropped from 0.7001 at 25 °C to 0.5717 at 35 °C and thereafter it followed a gradually decreasing trend by an increase in the temperature down to 0.5251 at 60 °C. Herein, A_n_ and N_0_ as the constants in Equation (9) were calculated as 0.048 and 775 K, respectively by linear fitting on the projection of *n* values on Ln (*n*) vs 1/*T* graph as shown in Figure 12a. Similarly, *k* values at any given temperature in our experimental range were evaluated by Equation (10) where *k*_1_ and *k*_2_ were calculated to be −4.13 × 10^−5^ gr/mm^2^. s^n^ and 3.86 × 10^−7^ gr/mm^2^·s^n^. K by line fitting on the *k* vs. *T* graph as shown in Figure 12b.

Table 2 summaries the *φ* vales calculated based on Equation (14) for ABS-UPR interphases obtained at different temperatures. It can be seen that the volume fraction of resin at the interface decreases by increasing the process temperature. This can be explained by a difference in the progress of diffusion front line which was accelerated by an increase in the temperature while such an increase in temperature led to a faster curing and, therefore, lower diffusion time limiting the volume of diffused resin in the interphase area.

### 3.5. Interphase’s Mechanical Response by Microhardness

**Figure 13:**
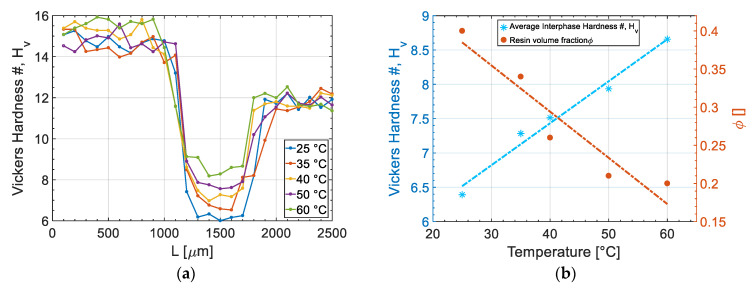
(**a**) Vickers microhardness values at the interphase vicinity for UPR-ABS interphase, and (**b**) correlation between interphase hardness and resin volume fraction.

Figure 13a shows the variations of Vickers microhardness numbers in the vicinity of the interphase prepared at different temperatures. It was observed that the hardness numbers at the interphase for all the samples were lower than for both pure UPR and ABS. It can also be seen that there was a transition region from the UPR to ABS (between 1000 µm and 1200 µm) as a sharp drop in the hardness value followed by a minimum value plateau indicating the interphase area and ended by an increase to ABS hardness value. The plateau lengths were correlated with interphase thicknesses obtained by the microscopic observations. The drop in the hardness at the interphase regions can be explained by the plasticizing effect of unreacted UPR monomers due to the limited access to reaction components at the interphase area. It can be seen from Figure 13a that the interphase hardness is correlated with the processed temperature science a higher cure temperature results in an increase in interphase hardness. 

To relate the microhardness at the interphase to the processing condition and microstructure of interphase, Figure 13b exhibits the averaged harness at the interphase (average of 1200 µm–1700 µm) and volume fraction of resin at the interphase (*φ*) calculated by Equation (14) on different processing temperatures. It can be seen that samples prepared at higher temperatures had a lower φ and hence a less resin presence at interphase to act as a plasticizer and consequently this resulted in a higher hardness values compared to specimens prepared at lower temperatures. On the other hand, samples prepared at lower temperatures showed a lower hardness due to higher volume fraction of resin at the interphase. Figure 13b provides a clear correlation on the process-microstructure and properties relation at the interphase of ABS with UPR.

## 4. Conclusions and Future Work

The interphase of a co-bonded ABS-UPR was investigated in this work by critically correlating the processing conditions, chemo-rheological behaviour, resulting microstructure and mechanical performance. The swelling of ABS with UPR was identified as a contributing parameter to the interphase size. It was observed that the interphase size changed with the processing temperature and it was correlated with thermal changes in the viscosity and cure kinetics of UPR through the gel time. The diffusivity of UPR into ABS was estimated by using the 1D inverse diffusion model in which the experimentally characterized temperature-dependent gelation time and interdiffusion thicknesses were utilized. The diffusivities obtained were fit to an Arrhenius relationship which can be used further for process simulations with various temperature ranges. More characterization experiments must be carried out to validate the diffusivities obtained and the developed diffusivity model. It was shown that the diffusivity was inversely proportional with the viscosity. The resin uptake experiments were employed to understand the kinetics of UPR diffusion into ABS at different temperatures. Accordingly, a resin uptake model was fitted to the experimentally obtained data by taking the processing temperature and cure kinetics of UR into account. The developed model was used to determine the volume fraction of the resin at the ABS-UPR interphase. The diffusivity coefficient obtained here is provide substantial information for future studies on the process modelling of multi-material composites and complex shaped hybrid structures. Microhardness tests showed that the hardness of interphase was lower than both ABS and UPR regions due to the plasticization effect of UPR molecules at the interphase. A correlation was created between the processing condition, resin volume fraction and its microhardness at the interphase which can be used for designing such a hybrid MMCs in the future. 

In the current work, the effect of the normal force used in the swelling experiments with the rheometer on the polymer interdiffusion at the ABS-UPR interface was not taken into account which is considered as a future work. Similarly, the effect of processing temperature on the viscosity up to the gelation is also defined as future work because the change in viscosity might influence the interphase thickness which was assumed to be negligible in this study.

## Figures and Tables

**Table 1:** Empirical rate constant (*k*), and transport exponent (*n*) for ABS-UPR at different temperatures.

Temperature (°C)	25	35	40	50	60
*n*	0.7002	0.5717	0.5538	0.5254	0.5251
*k* (gr/mm^2^·s^n^)	3.86 × 10^−7^	1.88 × 10^−6^	2.62 × 10^−6^	4.12 × 10^−6^	5.25 × 10^−6^

**Table 2:** Volume fraction of UPR (*φ*) at the ABS/ UPR interphase obtained from Equation (14).

Temperature (°C)	25	35	40	50	60
φ	0.40	0.34	0.26	0.21	0.20

## Data Availability

The data presented in this study are available on request from the corresponding authors. The data are not publicly available due to legal and privacy issues.
